# Encoding of mechanical nociception differs in the adult and infant brain

**DOI:** 10.1038/srep28642

**Published:** 2016-06-27

**Authors:** Lorenzo Fabrizi, Madeleine Verriotis, Gemma Williams, Amy Lee, Judith Meek, Sofia Olhede, Maria Fitzgerald

**Affiliations:** 1 Department of Neuroscience, Physiology & Pharmacology, University College London, Gower Street, London WC1 E6BT, UK; 2 Elizabeth Garrett Anderson Obstetric Wing, University College Hospital, London NW1 2BU, UK; 3 Department of Statistical Science, University College London, Gower Street, London WC1 E6BT, UK

## Abstract

Newborn human infants display robust pain behaviour and specific cortical activity following noxious skin stimulation, but it is not known whether brain processing of nociceptive information differs in infants and adults. Imaging studies have emphasised the overlap between infant and adult brain connectome architecture, but electrophysiological analysis of infant brain nociceptive networks can provide further understanding of the functional postnatal development of pain perception. Here we hypothesise that the human infant brain encodes noxious information with different neuronal patterns compared to adults. To test this we compared EEG responses to the same time-locked noxious skin lance in infants aged 0–19 days (n = 18, clinically required) and adults aged 23–48 years (n = 21). Time-frequency analysis revealed that while some features of adult nociceptive network activity are present in infants at longer latencies, including beta-gamma oscillations, infants display a distinct, long latency, noxious evoked 18-fold energy increase in the fast delta band (2–4 Hz) that is absent in adults. The differences in activity between infants and adults have a widespread topographic distribution across the brain. These data support our hypothesis and indicate important postnatal changes in the encoding of mechanical pain in the human brain.

The ability to perceive a noxious stimulus is essential for life, but for healthy infants the first painful experience does not occur until well after birth. Noxious sensory information is transmitted to the spinal cord^1^,^2^ and cerebral cortex in newborn human infants^3^,^4^, suggesting established ascending synaptic input from spinal mechanical nociceptive circuits. However, as yet, little is known about how neonatal cortical nociceptive circuits are organised or about the patterns of neural activity generated in the newborn human cortex by noxious stimulation.

Recent brain imaging studies have emphasised that the hallmark organizational functions of the human connectome are present by birth^5^,^6^,^7^. This is supported by the finding that those brain areas activated by experimental pinprick stimulation in the adult are also activated in the first postnatal week^8^. However, the postnatal period is also marked by changing patterns of neural activity across the developing cortex; for example, the posterior basic alpha rhythms (8–12 Hz) only emerge at 3–4 months^9^. Early synaptic connections are initially refined by spontaneously generated neuronal activity and later by sensory experience which adapts brain networks to environmental conditions^10^. Therefore, a better understanding of the maturation of neonatal cortical pain circuits in human infants can be gained by comparing the neural activity in the brain following early life noxious stimulation with that in adults.

In the adult, a brief peripheral noxious stimulus, such as a skin laser, electrical, or tissue-damaging stimulus, evokes a clear ERP (usually named N2-P2 because it consists of a negative (N)-positive (P) deflection), maximal at the vertex^11^,^12^, preceded by an earlier contralateral somatosensory evoked potential (SEP) representing the arrival of the first afferent volley to the somatosensory cortex (N1-P1). The vertex ERP has been used to study the effect of various physical, pharmacological, and cognitive interventions on nociceptive information processing. However, recent findings have highlighted the limitations of ERP analysis alone. Sensory stimulation may elicit energy changes at specific frequency bands that are not phase-locked to the stimulus and are therefore lost in the ERP averaging process^13^. For example, nociceptive C fiber input elicits a non-phase-locked alpha (8–12 Hz) energy decrease (event-related desynchronization, ERD) and a beta (15–30 Hz) energy increase (event related synchronization, ERS), which are only observable when a time-frequency decomposition approach is used^14^,^15^. While modulation of beta oscillations have traditionally been associated with movement, there is evidence that beta oscillations in sensorimotor cortex may serve large-scale communication between sensorimotor and other brain areas, which might be especially important for pain and pain expression^16^,^17^. Enhancement of gamma oscillations (30–100 Hz) over contralateral somatosensory cortex is related to subjective pain intensity^18^ and perhaps reflects the internal representations of salient stimuli that require enhanced or preferred processing. Moreover, cortical oscillations are likely to not only be a read-out of resonating neuronal activity, but may play a causal role in selective information transmission and, consequently, in conscious perception^19^.

Here we used a time-frequency decomposition approach to analyse the noxious-evoked electroencephalographic (EEG) activity in a group of healthy full-term infants. Using a time-locked, clinically required, skin breaking lance to trigger activity in the human infant cortex we analysed the cortical oscillations in the developing brain that are associated with early life noxious stimulation. The results were directly compared to the EEG activity evoked in a group of healthy adult volunteers receiving the same lance stimulus. The results show a clearly defined and distinct pattern of noxious evoked oscillations in the neonate, not seen in the adult. These are specific low frequency oscillations in the delta band (2–4 Hz) and may be important in shaping developing nociceptive networks in the human cortex.

## Methods

### Participants

Eighteen healthy full term 0–19 day old (5.8 ± 4.3, mean ± SD) infants (twelve males; born at 37–42 weeks gestational age) from the Elizabeth Garrett Anderson and Obstetric Hospital were included in this study. Medical charts were reviewed and, at the time of study, infants were assessed as clinically stable. Infants were not eligible for inclusion in the study if they were (1) receiving analgesics, sedatives or other psychotrophic agents; (2) showing signs of tissue damage on the lower limbs; (3) born to diabetic mothers or opioid users; (4) asphyxiated at birth or (5) born with congenital malformations or other genetic conditions. Informed written parental consent was obtained prior to each study. For all heel lances, standard hospital practice was followed: all infants were handled by trained health carers or by their parents and soothed as required.

For comparison, twenty-one healthy adult volunteers (four males) aged 23–48 years (29.7 ± 6.0, mean ± SD) participated in this study. All participants gave written informed consent.

The study conformed to the standards set by the Declaration of Helsinki and was approved by the Joint UCL and UCLH Research Ethics committees.

### EEG recording

Recording electrodes (disposable Ag/AgCl cup electrodes) were positioned according to the modified international 10/20 electrode placement system at Fp1, Fp2, F7, F8, Fz, Cz, CPz, C3, C4, CP3, CP4, T3, T4, T5, T6, O1 and O2. Recordings were referenced to FCz (all infants and eleven adults) or to linked ear lobes (ten adults) and were all re-referenced to Fz post-acquisition. The ground electrode was placed on the chest in all test occasions. Electrode/skin contact impedance was kept to a minimum by abrading the skin with EEG prepping gel and using EEG conductive paste. Electrodes were held in place with an elastic net and leads were tied together to minimise electrical interference. EEG activity, from DC (or 0.05 Hz in 6 infants) to 70 Hz, was recorded using the Neuroscan SynAmps2 EEG/EP recording system. Signals were digitised with a sampling rate of 2 kHz and a resolution of 24 bit.

### Experimental protocol

Infant studies were conducted on the hospital wards and lasted a maximum of one hour. The noxious cutaneous tissue damaging procedure was a clinically required heel lance performed to collect a blood sample. The lance was performed 8/18 times on the right foot and 10/18 times on the left foot. EEG recordings in response to stimulation on the right heel were flipped in respect to the midline (e.g. C3 swapped with C4) in order to have the contralateral side always on the same side of the scalp topography independently of the stimulation side. No heel lances were performed solely for the purpose of the study. Following the heel lance, the foot was not squeezed for a period of at least 30 s to ensure that the recorded responses were solely due to the lance. Skin wounds were dressed with cotton wool.

Adult studies were conducted in a quiet, temperature-controlled room purpose-built for research and lasted a maximum of one hour. Participants laid supine on a hospital bed for EEG recording. They were asked to relax their jaw (to reduce muscle artefact) and close their eyes (to reduce eyeblink artefact). The noxious cutaneous tissue-damaging procedure was a lance performed on the palmar surface of the distal phalanx of the fifth finger of the left hand using a sterile lancet (Tenderfoot, International Technidyne Corporation). The skin was wiped with antiseptic before lancing and wounds were dressed with cotton wool. Finger lances were considered painful by all participants, with an average pain score of 40.0 out of 100^12^.

### Time-locking and stimulus application

Lances were time-locked to the EEG recording using an accelerometer mounted to the superior surface of the lancet which detected the vibration caused by the blade release^20^. At least 2 mins prior to each lance, a control stimulus was applied at the same body location (fifth finger of the hand for the adults, heel for the infants). The lancet was rotated by 90° and placed against the body surface so that when the spring-loaded blade was released it did not contact the skin. Subjects experienced only the non-noxious tactile sensation and auditory click associated with the triggering of the blade.

### Data pre-processing

Continuous EEG data were segmented into epochs of 5 seconds, from 2 seconds pre-stimulus to 3 seconds post-stimulus. For the adult recordings, eye-blink artefacts were removed from contaminated trials in EEGLAB (http://sccn.ucsd.edu/eeglab), using independent component analysis (ICA). Independent components clearly representing eye-blinks in terms of topographical distribution and time course were low pass filtered at 20 Hz with a zero-phase 2^nd^ order Butterworth filter and then removed from the traces. In addition, epochs contaminated by gross movement artefacts (signal exceeding ±100 μV at any time point or larger than 2*SD of the baseline period for more than ¾ of the epoch length) were rejected. These rejection criteria were applied independently to each recording electrode so that an entire trial was not discarded if only few electrodes were contaminated.

### Event-related potential (ERP) analysis

Event related potential analysis was conducted at the vertex electrode Cz. EEG epochs were band pass filtered between 1–30 Hz with a zero-phase 2^nd^ order Butterworth filter and segmented into epochs of 1.5 s, from 0.5 s pre-stimulus to 1 s post-stimulus. Each epoch was baseline corrected using the pre-stimulus interval as a reference. Two infant lance epochs were rejected due to movement artefact; the corresponding control epochs were also rejected to allow paired comparisons. In order to correct for inter-subjects latency jitter, traces were aligned by Woody filtering between 0–400 ms after stimulation (allowed jitter for correction: −50 to +50 ms) for the adult group and between 50–300 ms after stimulation (allowed jitter for correction: −20 to +20 ms) for the infant group^21^,^22^. The resulting latency shifts were applied to all the other recording electrodes.

Four separate average event-related potential (ERP) waveforms were computed for each age group (infant and adult) and stimulus modality (control and noxious). Peaks at Cz were identified from these group averages and scalp topography maps were calculated. In 9 infants a reduced number of electrodes were used due to limited access to the scalp or parental request. In these cases the missing electrode recordings were estimated by a spherical interpolation of the others^23^.

To assess the differences between the ERP waveforms, repeated measures ANOVA was conducted for each time point of the epoch (within variable: stimulus modality; between variable: age group). The results were then further investigated with post-hoc t-testing. Paired samples t-tests were conducted for each time point of the epoch across stimulus modality within an age group. Independent samples t-tests were instead conducted to assess the difference in the responses to control and noxious stimulation between infants and adults. The significance threshold was assumed to be α = 0.05, however, because these tests were conducted at any time point t in time, false discovery rate was used to correct for multiple comparisons^24^. The total number of independent tests performed is related to the length of the smoothing filter used. Because we used a 1–30 Hz band-pass filter, we considered 30 independent tests per second in the multiple comparisons correction. This point-by-point analysis is generally used to identify differences in signal amplitude^25^, but when comparing different age groups may also be affected by differences in latency or signal slopes. For this reason we also compared the peak amplitudes independently of their latencies using repeated measures ANOVA.

### Time-frequency analysis

EEG epochs were high-pass filtered at 0.5 Hz with a zero-phase 2^nd^ order Butterworth filter and re-segmented into epochs of 5 s, from 2 s pre-stimulus to 3 s post-stimulus. The same latency shifts calculated with Woody filtering in the ERP analysis were applied to each trial. . Eleven epochs were rejected due to movement artefacts (1 adult lance, 1 adult control, 6 infant lances and 1 infant control epochs). The larger number of rejected epochs in the time-frequency analysis compared to the ERP analysis is due to late artefacts occurring after the ERP, but within 3 seconds post stimulus. In the accepted epochs, behavioural responses (e.g. crying, head movements) either occurred after 3 seconds post stimulus or did not appear to affect recording at Cz. A complex time-frequency spectral estimate W(a, b) of the remaining epochs recorded at Cz was calculated at each point (a, b) of the time-frequency plane (from 2 sec pre-stimulus to 3 sec post-stimulus in the time domain, and between 0.5–70 Hz (in logarithmic steps) in the frequency domain) using a complex Morse wavelet transform^26^. We estimated the stimulus-induced energy changes time-locked to lance and control for infants and adults separately and then we compared these patterns of evoked activity across stimulus type and age group.

The energy changes in the EEG that were induced by the stimuli were estimated as a group median. This was done by calculating the energy (i.e. modulus square) of the TF transform for each individual trial and then taking the sample median at each time-frequency point (a, b). This quantity was then normalised by the mean energy content of the baseline period (−1000 to −500 ms before stimulus) at each frequency, representing the percentage energy changes phase- or non-phase-locked to the stimulus. In order to compare the patterns of evoked activity between adults and infants or between lance and control, we conducted a group comparison as described elsewhere^12^, but substituting the mean estimator with a median estimator. We chose a median instead of a mean estimator to make the estimation less susceptible to the presence of outliers, but this meant that we could not use standard statistical tests and needed to derive the correct modelling distribution from first principles (Supplementary Methods).

The significance threshold was assumed to be α = 0.05, however, because these tests were conducted at any point (a, b) of the time-frequency plane, false discovery rates was used to correct for multiple comparisons^24^. The total number of independent tests performed was estimated in two steps: (i) calculating the number of independent tests at each frequency by dividing the length of the considered epoch by the length of the wavelet at that frequency (accounting for the correlation caused by the smoothing in time of the wavelet transform); (ii) adding those numbers across frequencies.

The areas of the time-frequency plane where the difference between two groups was significant were identified as regions of interest (ROIs) at Cz. To define the topographical distribution of these differences the same time-frequency transformation described above was conducted for each scalp electrode. The topographic map of each ROI was calculated by averaging the difference between two groups within the significance boundaries at Cz and at the same time-frequency patch for all other electrode site.

## Results

### Noxious stimulation evokes a distinct ERP in infants which is not present in adults

**Figure 1:**
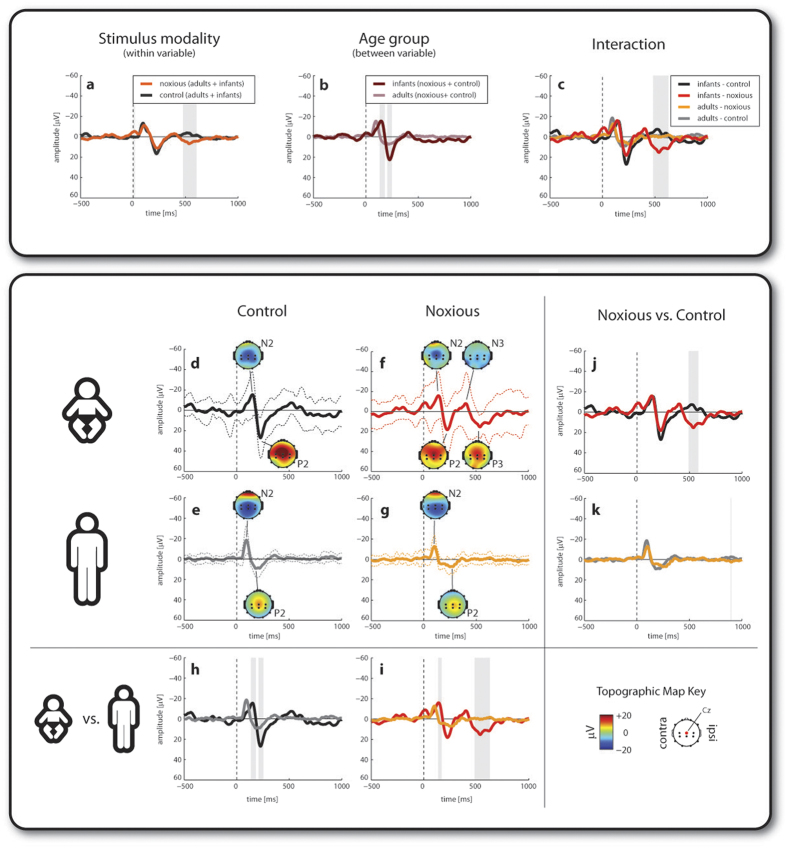
Event-related potentials (ERP) following a control and a skin-breaking noxious stimulus in full term infants and adults. (**a–c**) Results of the point-by-point repeated measures ANOVA: stimulus modality main effect; age group main effect and interaction. (**d–g**) Group averages (±SD) and (**h–k**) results of the point-by-point paired (within age group) and unpaired (across age groups) t-tests. Statistical significance after correction for multiple comparisons is represented by shaded areas.

Skin-breaking noxious stimulation evoked specific late event related potentials (ERPs) in newborn infants^3^,^4^. Figure 1 shows that noxious and control stimulation evoked a negative-positive waveform (a negative peak, N2, followed by a positive peak, P2) in infants and adults, but the noxious stimulation also evoked a second wave (N3-P3) in infants which was completely absent in adults.

The earlier N2-P2 waveform occurred between 100–300 ms and had a symmetrical distribution maximal at the vertex (Fig. 1d–g). This was not significantly different in amplitude between noxious and control stimulation (repeated measure ANOVA: N2 – p = 0.48; P2 – p = 0.09), while the P2 peak was larger in infants compared to adults (N2 – p = 0.99; P2 – p = 0.001). Table 1 and point-by-point comparisons of the ERP waveforms show also that the peaks occurred later in infants than in adults (Fig. 1b,h,i).

The later N3-P3 waveform had also a symmetrical distribution maximal at the vertex and was only detectable following noxious stimulation in the infant group average (Fig. 1f). Accordingly, point-by-point comparisons of the ERP waveforms revealed: (i) significant interaction between stimulus type and age group (Fig. 1c) and (ii) differences in the time interval 500–700 ms between the two age groups following noxious stimulation (Fig. 1i) and between the control and noxious stimulus in infants (Fig. 1j).

**Figure 2:**
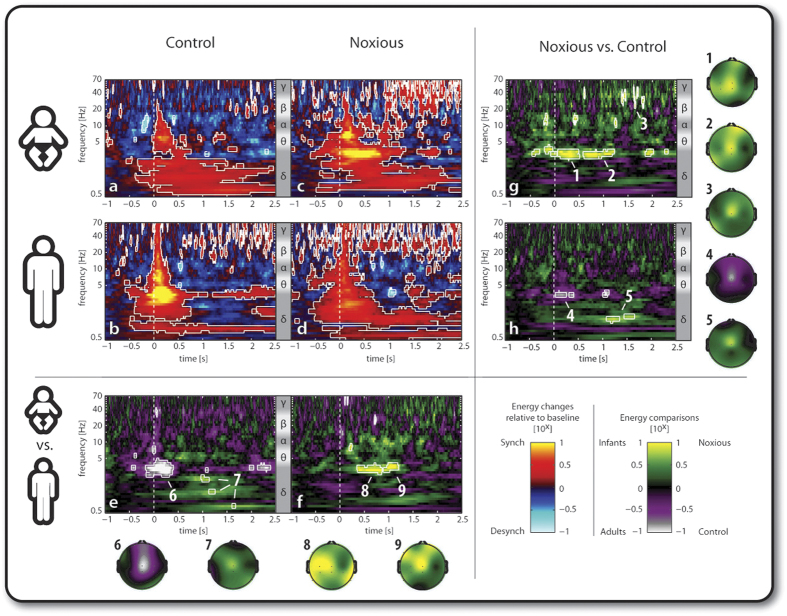
Time-frequency decomposition of the EEG responses at Cz to a control and a skin-breaking noxious stimulus in full term infants and adults. (**a–d**) Median energy changes relative to baseline (−1 to −0.5 s). The colour scale is logarithmic: positive (red-yellow) and negative (blue-light blue) values respectively represent energy increases (signal synchronization) and decreases (signal desynchronization) compared to baseline. Circumscribed areas represent significant evoked activity compared to baseline. (**e,f**) Energy comparisons between infants and adults for noxious and control stimulus. The colour scale is logarithmic: positive (green-yellow) values represent larger energy for the infants while negative (purple-white) values represent larger energy for the adults. Circumscribed areas represent significant evoked activity differences between the two age groups. (**g,h**) Energy comparisons between noxious and control stimulus within the same age group. The colour scale is logarithmic: positive (green-yellow) values represent larger energy following noxious stimulation while negative (purple-white) values represent larger energy following control stimulation. Circumscribed areas represent significant evoked activity differences between the two stimuli. An approximated EEG frequency band division is displayed next to each time-frequency plot. Time-frequency regions of interest (ROIs) have been labelled and their topographical distribution is displayed to the right and at the bottom of the figure.

While the ERP analysis demonstrates that the N3-P3 is a noxious and age specific waveform, it does not determine which brain activity patterns are involved in the stimulus responses. Time-frequency analysis was then employed to address this question. This approach revealed further differences in stimulus related activity between infants and adults and between control and lance stimulation in distinct time-frequency regions of interest at Cz (ROIs in Fig. 2). In the text, ROI data are presented as mean ratios within a given ROI. A ratio larger than 1:1 (e.g. 2:1) means that the energy within the ROI is larger for the first group in the comparison while a ratio smaller than 1:1 (e.g. 1:2) means that the energy is larger for the second group in the comparison. Groups have been labelled as: IC = infant control; IN = infant noxious; AC = adult control; AN = adult noxious.

### Control stimulation evokes a more synchronised response in adults than in infants

Figure 2a–f illustrates the data in the time frequency domain. Figure 2a shows that control stimulation in infants evoked long duration (up to 2.5 sec post-stimulus) energy increases in the low-medium frequency bands (0.5–15 Hz) of up to 7.5 times over baseline (Fig. 2a). The adult control response (Fig. 2b) did not display this long duration energy increase but was highly synchronous, short lasting and spanned a wide frequency range (0.5–70 Hz) (Fig. 2b). These differences are marked as ROI 6 (1:9 (IC:AC), 0–400 ms) and ROI 7 (5:1 (IC:AC), 950–1650 ms) in Fig. 2e, where the infant and adult control responses are compared.

### Noxious stimulation evokes gamma oscillations in both infants and adults

Figure 2c shows that noxious stimulation in infants evoked a strong ultra-late beta-gamma energy increase with a delayed onset (0.9 sec), which was not evoked by the control stimulus (Fig. 2a). This difference is marked as ROI 3 (7:1, 1350–1850 ms) in Fig. 2g, where the infant responses to noxious and control stimulation are compared. Figure 2d shows that gamma oscillations are also evoked by noxious stimulation in adults and but that when noxious and control stimulati in adults are compared the differences are greatest in lower frequency bands (Fig. 2h: ROI 4–1:7 (AN:AC), 30–400 ms; ROI 5–6:1 (AN:AC), 1070–1650 ms).

### Noxious stimulation evokes a distinct increase in fast delta activity in neonates, but not in adults

Noxious stimulation in infants evoked long duration (up to 2.5 sec post-stimulus) energy increases of up to 18 times compared to baseline in the low-medium frequency bands (0.5–15 Hz; Fig. 2c). This is phase-locked to the stimulus up to 750 ms, therefore underlying the ERPs, and is not phase-locked afterwards (Supplementary Figure 1). Figure 2d shows that, in adults, the energy increase in response to the same stimulus spanned the whole frequency range (0.5–70 Hz), but was weaker (up to 6.6 times) and shorter in duration in the fast delta (2–4 Hz) and theta band (4–8 Hz) compared to infants (0.8 sec post-stimulus in adults against 1.4 sec in infants). These differences can be seen in Fig. 2f, where the infant and adult responses to noxious stimulation are compared. Noxious stimulation in infants evokes a significantly larger initial energy increase in the fast delta band (ROI 8–8:1 (IN:AN), 350–850 ms) compared to adults; in addition, this increase is sustained for a longer period of time (ROI 9–11:1 (IN:AN), 900–1200 ms) in the infants. These patterns were specific to the infant nociceptive response and were also observed in within infant (Fig. 2g: ROI 1–8:1 (IN:IC), 50–500 ms and ROI 2–9:1 (IN:IC), 600–1200 ms) but not in within adult (Fig. 2h) comparisons or control comparisons (Fig. 2e).

### Spatial localisation of noxious and non-noxious brain activity is conserved within but not between age groups

Figure 2 shows the topographical distribution of the described differences within age (ROI 1–5) and between ages (ROI 6–9). The energy differences between lance and control at the same age are largely localised around the vertex (ROIs 1–5), but those between infants and adults are more diffuse throughout the scalp (ROIs 6–9).

## Discussion

In this study we have identified distinct functional activity patterns specific to neonatal nociceptive cortical networks by comparing the cortical responses to the same cutaneous tissue damage in infants and adults. The noxious, skin-breaking lance stimulus evokes clear responses in both the infant and adult human brain. These responses have some similarities, but also clear differences. The event related potential (ERP) and gamma oscillations, characteristic of adult sensory responses, are already present at birth, although at longer latencies. However, noxious stimulation in infants evokes a distinct ERP that is entirely absent in adult and is underpinned by an energy increase in the fast delta frequency. These results support our hypothesis that even though the basic organizational functions of the human brain are already in place at birth^5^,^6^,^7^,^27^, there are fundamental differences in the way the neonatal brain encodes noxious stimulation compared to adults.

Noxious and innocuous stimulation evoke a typical N2-P2 ERP between 100–300 ms post stimulation that is maximal at the vertex in infants and adults. This response has been suggested to arise from the large diameter, myelinated Aβ tactile and Aδ nociceptive input^12^,^15^. The decrease in latency between the infant and adult N2-P2 observed here is likely to reflect the increase in myelination and the decreased synaptic delay throughout the somatosensory nervous system^28^,^29^,^30^. The longer delays in infants also explain the weaker and longer lasting synchronization induced by the control stimulus in infants. The ultra-late energy increase following noxious stimulation in the slow delta band in adults is likely to be associated to slowly conducting nociceptive C fibre activation^25^,^31^. The N2-P2 ERP was not significantly different between control and lance and the associated energy increase was larger following control in adults. This is probably because this ERP is affected by the saliency of the incoming stimulus^32^,^33^,^34^, which is stronger for the first stimulus of a series. This was the case for the control stimulus which was always performed before the lance. This strong saliency effect is likely to be absent in neonates as the development of associative networks appears to lag behind that of the primary sensory cortices^35^. This would make the N2-P2 and its associated energy increase more directly related to stimulus intensity in infants, explaining the larger initial energy change following lance compared to control in this age group.

Noxious stimulation evoked gamma frequency oscillations in infants and adults. This pattern had an ultra-late onset in infants, while in adults it started immediately after stimulation and was sustained for a long time period (2.5 sec). Such oscillations were also present, to a weaker extent, following control stimulation in adults. Gamma oscillations predict the pain perceived by the subject under experimental laser stimuli^18^, however they have also been observed following non-nociceptive somatosensory stimuli^36^,^37^,^38^,^39^. Overall oscillatory activity in the gamma range could represent the integration of low-level cortical representations of physical stimulus qualities in the primary somatosensory cortex, such as position and intensity, with higher-level cognitive functions, such as attention and anticipation^40^,^41^. In adults this allows processes such as reinforcement-based learning through prediction errors^42^, endogenous analgesia through spatial and temporal filtering of nociceptive information^43^, or perception modulation through attention redirection^44^,^45^. It is difficult, however, to extrapolate these mechanisms to the infant brain.

The presence of the N2-P2 ERP and of the gamma oscillations following noxious stimulation in neonates indicates that the nociceptive information reaches the primary sensory centres and is forwarded to other brain regions through similar routes to the adult, only with longer latencies. However, our results show that there are also fundamental differences in the way that the neonatal brain encodes noxious information. Noxious stimulation evokes a specific ultra-late ERP that is completely absent in adults (N3-P3). Here we have used time-frequency decomposition to show that this ultra-late ERP corresponds to a very strong energy increase in the delta band. Delta frequency activity is a typical feature of the developing brain^46^ and a key component of the delta brushes, which are synchronised neuronal bursts thought to be critical in establishing the initial cortical networks involved in sensory processing^3^,^47^,^48^. These can be generated by endogenous activity or evoked by peripheral sensory stimulation, allowing for cortical functional refinement and segregation^10^ underlying the transition from immature to more defined cortical responses. It has been proposed that these patterns are generated by the subplate, a transient brain structure below the cortical plate which is prominent in early development and dissolves with maturation^49^. While other sensory systems such as the visual, somatosensory, and auditory are activated almost continually from before birth, the nociceptive system develops in the absence of such patterned afferent inputs. The delta pattern observed here could therefore represent the activation of subplate neurons which are still present in the early postnatal period and, as a result, play a role in the experience-dependent postnatal shaping of nociceptive circuits^50^,^51^,^52^.

Our finding of a widespread topographic distribution of differences between infants and adults in both noxious and control evoked oscillatory activity may also result from this postnatal cortical organization^53^,^54^. Cortical functions in infants appear to engage wider cortical areas and evoke broader interactions across brain regions compared to adults^8^,^55^,^56^. This is consistent with animal data where somatosensory cortical maps undergo experience driven refinement during a postnatal critical period^57^.

There are limitations to this study. The stimulus site in adults and neonates is not the same, but was chosen to be comparable in skin thickness, innervation density and relative size rather than actual position. These are also the site used clinically for blood sampling. Since this study is about pain processing rather than sensory discrimination, actual stimulus site is less important. The gender balance of the adults and neonates was also not the same but since sex differences in responses to experimental pain in both children and adults are far from clear^58^,^59^, this too is unlikely to have a great impact on the data. Difference in stimulation site, but not in sex distribution, between infants and adults could contribute to the differences in N2-P2 latency and amplitude across age groups observed here^60^,^61^; however these differences cannot explain the gamma activity rhythms and the ultra-late nociceptive ERP and associated delta band energy increase in neonates observed in this study. In addition, although this was not a focus of this study, flipping the hemispheres for analysis may have blunted any potential lateralised responses.

Even though Laplacian or a Common Average ERP referencing is widely used in adults, referencing to midline frontal electrodes is the standard recommended for neonatal EEG^62^,^63^.The Laplacian scheme needs ~64 electrodes^64^ which is not feasible for infants in neonatal units. The Common Average scheme is also not recommended in neonates because of too few electrodes and the large spontaneous potentials in some electrodes affect the average^46^.

The basic layout of neuronal connections in the developing cortex is first established through molecular factors that guide axons to their target areas and restrict connectivity to defined sub-populations^65^. These synaptic connections are then further refined by spontaneously generated neuronal activity^10^. Finally, postnatal sensory experience shapes brain networks according to the environment^57^. Consistent with this, we have previously shown that cortical responses to noxious stimuli in preterm infants develop from non-specific neuronal bursts into modality selective event related potentials^3^, which are still present at 1 year of age^66^. In this study we have provided new insight into these infant nociceptive potentials, showing that they are composed of high energy and prolonged oscillations within the fast delta band. This noxious evoked activity is restricted to the immature cortex and may represent the mechanism by which noxious inputs begin to shape nociceptive specific networks out of a more diffuse somatosensory connectome.

## Additional Information

**How to cite this article**: Fabrizi, L. *et al*. Encoding of mechanical nociception differs in the adult and infant brain. *Sci. Rep.* **6**, 28642; doi: 10.1038/srep28642 (2016).

## Supplementary Material

Supplementary Information

## Figures and Tables

**Table 1:** Event Related Potential amplitudes and latencies.

		Control	Noxious
**Infants**	N2	−15.4 ± 21.5 μV	−16 ± 23.1 μV
		*151.5* ms	*140* ms
	P2	*227* ms	*225.5* ms
		27.0 ± 19.2 μV	18.2 ± 22.8 μV
	Pk-to-Pk	42.4 ± 35.3 μV	34.2 ± 27.6 μV
	N3	/	−8 ± 29.5 μV
			*403* ms
	P3	/	15.4 ± 11.3 μV
			*538* ms
	Pk-to-Pk	/	23.4 ± 33.5 μV
**Adults**	N2	−18.6 ± 13.4 μV	−12.8 ± 10.6 μV
		*93.5* ms	*102* ms
	P2	*180.5* ms	*249.5* ms
		9.3 ± 7.9 μV	7.2 ± 6.9 μV
	Pk-to-Pk	27.8 ± 16.5 μV	20 ± 15.5 μV

Group averaged (±SD) peak amplitudes and latencies and peak-to-peak amplitudes of the vertex event related potentials (ERP) following a control and a skin-breaking noxious stimulus in full term infants (n = 16) and adults (n = 21). The second waveform is only present following noxious stimulation in infants.

## References

[b1] Andrews K. & Fitzgerald M. Flexion reflex responses in biceps femoris and tibialis anterior in human neonates. Early Hum. Dev. 57, 105–110 (2000). 10735457 10.1016/s0378-3782(99)00059-6

[b2] Cornelissen L. . Postnatal temporal, spatial and modality tuning of nociceptive cutaneous flexion reflexes in human infants. PLoS One 8, e76470, 10.1371/journal.pone.0076470 (2013). 24124564 PMC3790695

[b3] Fabrizi L. . A shift in sensory processing that enables the developing human brain to discriminate touch from pain. Curr. Biol. 21, 1552–1558, 10.1016/j.cub.2011.08.010 (2011). 21906948 PMC3191265

[b4] Slater R. . Evoked potentials generated by noxious stimulation in the human infant brain. Eur J Pain 14, 321–326, 10.1016/j.ejpain.2009.05.005 (2010). 19481484

[b5] Doria V. . Emergence of resting state networks in the preterm human brain. Proc. Natl. Acad. Sci. USA 107, 20015–20020, 10.1073/pnas.1007921107 (2010). 21041625 PMC2993415

[b6] Toulmin H. . Specialization and integration of functional thalamocortical connectivity in the human infant. Proc. Natl. Acad. Sci. USA 112, 6485–6490, 10.1073/pnas.1422638112 (2015). 25941391 PMC4443373

[b7] van den Heuvel M. P. . The Neonatal Connectome During Preterm Brain Development. Cereb. Cortex 25, 3000–3013, 10.1093/cercor/bhu095 (2015). 24833018 PMC4537441

[b8] Goksan S. . fMRI reveals neural activity overlap between adult and infant pain. Elife 4, 10.7554/eLife.06356 (2015). PMC4402596 25895592

[b9] Niedermeyer E. In Electroencephalography: Basic Principles, Clinical Applications and Related Fields (eds Niedermeyer E. & Da Silva F. L.) (Lippincott Williams & Wilkins, 2005).

[b10] Hanganu-Opatz I. L. Between molecules and experience: role of early patterns of coordinated activity for the development of cortical maps and sensory abilities. Brain Res Rev 64, 160–176, 10.1016/j.brainresrev.2010.03.005 (2010). 20381527

[b11] Baumgartner U., Greffrath W. & Treede R. D. Contact heat and cold, mechanical, electrical and chemical stimuli to elicit small fiber-evoked potentials: Merits and limitations for basic science and clinical use. Neurophysiol. Clin. 42, 267–280, 10.1016/j.neucli.2012.06.002 (2012). 23040698

[b12] Fabrizi L. . Cortical activity evoked by an acute painful tissue-damaging stimulus in healthy adult volunteers. J. Neurophysiol. 109, 2393–2403, 10.1152/jn.00990.2012 (2013). 23427303 PMC3652217

[b13] Mouraux A. & Iannetti G. D. Across-trial averaging of event-related EEG responses and beyond. Magn. Reson. Imaging 26, 1041–1054, 10.1016/j.mri.2008.01.011 (2008). 18479877

[b14] Domnick C., Hauck M., Casey K. L., Engel A. K. & Lorenz J. C-fiber-related EEG-oscillations induced by laser radiant heat stimulation of capsaicin-treated skin. J Pain Res 2, 49–56 (2009). 21197293 10.2147/jpr.s4860 PMC3004625

[b15] Mouraux A., Guerit J. M. & Plaghki L. Non-phase locked electroencephalogram (EEG) responses to CO2 laser skin stimulations may reflect central interactions between A partial partial differential- and C-fibre afferent volleys. Clin. Neurophysiol. 114, 710–722 (2003). 12686279 10.1016/s1388-2457(03)00027-0

[b16] Senkowski D., Kautz J., Hauck M., Zimmermann R. & Engel A. K. Emotional facial expressions modulate pain-induced beta and gamma oscillations in sensorimotor cortex. J. Neurosci. 31, 14542–14550, 10.1523/JNEUROSCI.6002-10.2011 (2011). 21994371 PMC6703391

[b17] Kilavik B. E., Zaepffel M., Brovelli A., MacKay W. A. & Riehle A. The ups and downs of beta oscillations in sensorimotor cortex. Exp. Neurol. 245, 15–26, 10.1016/j.expneurol.2012.09.014 (2013). 23022918

[b18] Zhang Z. G., Hu L., Hung Y. S., Mouraux A. & Iannetti G. D. Gamma-band oscillations in the primary somatosensory cortex–a direct and obligatory correlate of subjective pain intensity. J. Neurosci. 32, 7429–7438, 10.1523/JNEUROSCI.5877-11.2012 (2012). 22649223 PMC6703598

[b19] Helfrich R. F. . Selective modulation of interhemispheric functional connectivity by HD-tACS shapes perception. PLoS Biol 12, e1002031, 10.1371/journal.pbio.1002031 (2014). 25549264 PMC4280108

[b20] Worley A., Fabrizi L., Boyd S. & Slater R. Multi-modal pain measurements in infants. J. Neurosci. Methods 205, 252–257, 10.1016/j.jneumeth.2012.01.009 (2012). 22285660 PMC3465552

[b21] Bromm B. & Scharein E. Principal component analysis of pain-related cerebral potentials to mechanical and electrical stimulation in man. Electroencephalogr. Clin. Neurophysiol. 53, 94–103 (1982). 6173204 10.1016/0013-4694(82)90109-2

[b22] Woody C. D. Characterization of an Adaptive Filter for Analysis of Variable Latency Neuroelectric Signals. Medical & Biological Engineering 5, 539-&, 10.1007/Bf02474247 (1967).

[b23] Delorme A. & Makeig S. EEGLAB: an open source toolbox for analysis of single-trial EEG dynamics including independent component analysis. J. Neurosci. Methods 134, 9–21, 10.1016/j.jneumeth.2003.10.009 (2004). 15102499

[b24] Benjamini Y. & Hochberg Y. Controlling the False Discovery Rate - a Practical and Powerful Approach to Multiple Testing. Journal of the Royal Statistical Society Series B-Methodological 57, 289–300 (1995).

[b25] Jankovski A., Plaghki L. & Mouraux A. Reliable EEG responses to the selective activation of C-fibre afferents using a temperature-controlled infrared laser stimulator in conjunction with an adaptive staircase algorithm. Pain 154, 1578–1587, 10.1016/j.pain.2013.04.032 (2013). 23707267

[b26] Olhede S. C. & Walden A. T. Generalized morse wavelets. IEEE Transactions on Signal Processing 50, 2661–2670, 10.1109/Tsp.2002.804066 (2002).

[b27] Allievi A. G. . Maturation of Sensori-Motor Functional Responses in the Preterm Brain. Cereb. Cortex, 10.1093/cercor/bhv203 (2015). PMC4677983 26491066

[b28] Brody B. A., Kinney H. C., Kloman A. S. & Gilles F. H. Sequence of Central-Nervous-System Myelination in Human Infancy.1. An Autopsy Study of Myelination. J. Neuropathol. Exp. Neurol. 46, 283–301, 10.1097/00005072-198705000-00005 (1987). 3559630

[b29] Gutrecht J. A. & Dyck P. J. Quantitative Teased-Fiber and Histologic Studies of Human Sural Nerve during Postnatal Development. J. Comp. Neurol. 138, 117-&, 10.1002/cne.901380109 (1970). 5412716

[b30] Cracco J. B., Cracco R. Q. & Stolove R. Spinal Evoked-Potential in Man - Maturational Study. Electroencephalogr. Clin. Neurophysiol. 46, 58–64 (1979). 88331 10.1016/0013-4694(79)90049-x

[b31] Hu L., Cai M. M., Xiao P., Luo F. & Iannetti G. D. Human brain responses to concomitant stimulation of adelta and C nociceptors. J. Neurosci. 34, 11439–11451, 10.1523/JNEUROSCI.1355-14.2014 (2014). 25143623 PMC6615513

[b32] Iannetti G. D. & Mouraux A. From the neuromatrix to the pain matrix (and back). Exp. Brain Res. 205, 1–12, 10.1007/s00221-010-2340-1 (2010). 20607220

[b33] Legrain V., Iannetti G. D., Plaghki L. & Mouraux A. The pain matrix reloaded: a salience detection system for the body. Prog. Neurobiol. 93, 111–124, 10.1016/j.pneurobio.2010.10.005 (2011). 21040755

[b34] Ronga I., Valentini E., Mouraux A. & Iannetti G. D. Novelty is not enough: laser-evoked potentials are determined by stimulus saliency, not absolute novelty. J. Neurophysiol. 109, 692–701, 10.1152/jn.00464.2012 (2013). 23136349 PMC3567386

[b35] Fransson P. . Early development of spatial patterns of power-law frequency scaling in FMRI resting-state and EEG data in the newborn brain. Cereb. Cortex 23, 638–646, 10.1093/cercor/bhs047 (2013). 22402348

[b36] Bauer M., Oostenveld R., Peeters M. & Fries P. Tactile spatial attention enhances gamma-band activity in somatosensory cortex and reduces low-frequency activity in parieto-occipital areas. J. Neurosci. 26, 490–501, 10.1523/JNEUROSCI.5228-04.2006 (2006). 16407546 PMC6674422

[b37] Fukuda M. . Short-latency median-nerve somatosensory-evoked potentials and induced gamma-oscillations in humans. Brain 131, 1793–1805, 10.1093/brain/awn100 (2008). 18508784 PMC2538581

[b38] Gaetz W. & Cheyne D. Localization of sensorimotor cortical rhythms induced by tactile stimulation using spatially filtered MEG. Neuroimage 30, 899–908, 10.1016/j.neuroimage.2005.10.009 (2006). 16326116

[b39] Rossiter H. E., Worthen S. F., Witton C., Hall S. D. & Furlong P. L. Gamma oscillatory amplitude encodes stimulus intensity in primary somatosensory cortex. Front Hum Neurosci 7, 362, 10.3389/fnhum.2013.00362 (2013). 23874282 PMC3711008

[b40] Hauck M., Lorenz J. & Engel A. K. Attention to painful stimulation enhances gamma-band activity and synchronization in human sensorimotor cortex. J. Neurosci. 27, 9270–9277, 10.1523/JNEUROSCI.2283-07.2007 (2007). 17728441 PMC6673131

[b41] van Ede F., Szebenyi S. & Maris E. Attentional modulations of somatosensory alpha, beta and gamma oscillations dissociate between anticipation and stimulus processing. Neuroimage 97, 134–141, 10.1016/j.neuroimage.2014.04.047 (2014). 24769186

[b42] Roy M. . Representation of aversive prediction errors in the human periaqueductal gray. Nat. Neurosci. 17, 1607–1612, 10.1038/nn.3832 (2014). 25282614 PMC4213247

[b43] Nahman-Averbuch H. . Distinct brain mechanisms support spatial vs temporal filtering of nociceptive information. Pain 155, 2491–2501, 10.1016/j.pain.2014.07.008 (2014). 25047783 PMC4250429

[b44] Bantick S. J. . Imaging how attention modulates pain in humans using functional MRI. Brain 125, 310–319 (2002). 11844731 10.1093/brain/awf022

[b45] Miron D., Duncan G. H. & Bushnell M. C. Effects of attention on the intensity and unpleasantness of thermal pain. Pain 39, 345–352 (1989). 2616184 10.1016/0304-3959(89)90048-1

[b46] Andre M. . Electroencephalography in premature and full-term infants. Developmental features and glossary. Neurophysiol. Clin. 40, 59–124, 10.1016/j.neucli.2010.02.002 (2010). 20510792

[b47] Colonnese M. T. . A conserved switch in sensory processing prepares developing neocortex for vision. Neuron 67, 480–498, 10.1016/j.neuron.2010.07.015 (2010). 20696384 PMC2946625

[b48] Milh M. . Rapid cortical oscillations and early motor activity in premature human neonate. Cereb. Cortex 17, 1582–1594, 10.1093/cercor/bhl069 (2007). 16950867

[b49] Vanhatalo S. & Kaila K. Development of neonatal EEG activity: from phenomenology to physiology. Semin Fetal Neonatal Med 11, 471–478, 10.1016/j.siny.2006.07.008 (2006). 17018268

[b50] Kostovic I. & Jovanov-Milosevic N. The development of cerebral connections during the first 20–45 weeks’ gestation. Semin Fetal Neonatal Med 11, 415–422, 10.1016/j.siny.2006.07.001 (2006). 16962836

[b51] Kostovic I. . Perinatal and early postnatal reorganization of the subplate and related cellular compartments in the human cerebral wall as revealed by histological and MRI approaches. Brain Struct Funct 219, 231–253, 10.1007/s00429-012-0496-0 (2014). 23250390

[b52] Kostovic I. & Judas M. The development of the subplate and thalamocortical connections in the human foetal brain. Acta Paediatr. 99, 1119–1127, 10.1111/j.1651-2227.2010.01811.x (2010). 20367617

[b53] Mitrukhina O., Suchkov D., Khazipov R. & Minlebaev M. Imprecise whisker map in the neonatal rat barrel cortex. Cereb. Cortex, 10.1093/cercor/bhu169 (2014). 25100857

[b54] Seelke A. M., Dooley J. C. & Krubitzer L. A. The emergence of somatotopic maps of the body in S1 in rats: the correspondence between functional and anatomical organization. PLoS One 7, e32322, 10.1371/journal.pone.0032322 (2012). 22393398 PMC3290658

[b55] Gao W. . Evidence on the emergence of the brain’s default network from 2-week-old to 2-year-old healthy pediatric subjects. Proc. Natl. Acad. Sci. USA 106, 6790–6795, 10.1073/pnas.0811221106 (2009). 19351894 PMC2672537

[b56] Johnson M. H. Functional brain development in infants: elements of an interactive specialization framework. Child Dev. 71, 75–81 (2000). 10836560 10.1111/1467-8624.00120

[b57] Erzurumlu R. S. & Gaspar P. Development and critical period plasticity of the barrel cortex. Eur. J. Neurosci. 35, 1540–1553, 10.1111/j.1460-9568.2012.08075.x (2012). 22607000 PMC3359866

[b58] Boerner K. E. . Sex differences in experimental pain among healthy children: a systematic review and meta-analysis. *PAIN* 155(5), 983–993 (2014). 10.1016/j.pain.2014.01.031 24508752

[b59] Racine M. . A systematic literature review of 10 years of research on sex/gender and experimental pain perception–Part 1: Are there really differences between women and men? *PAIN* 153(3), 602–618 (2012). 10.1016/j.pain.2011.11.025 22192712

[b60] Kakigi R., Watanabe S. & Yamasaki H. Pain-Related somatosensory evoked potentials. J. Clin. Neurophysiol. 17, 295–308 (2000). 10928641 10.1097/00004691-200005000-00007

[b61] Truini A. . Laser-evoked potentials: normative values. Clin. Neurophysiol. 116, 821–826, 10.1016/j.clinph.2004.10.004 (2005). 15792891

[b62] Korotchikova I., Stevenson N. J., Livingstone V., Ryan C. A. & Boylan G. B. Sleep-wake cycle of the healthy term newborn infant in the immediate postnatal period. Clin. Neurophysiol., 10.1016/j.clinph.2015.12.015 (2015). 26790580

[b63] Walls-Esquivel E., Vecchierini M. F., Heberle C. & Wallois F. Electroencephalography (EEG) recording techniques and artefact detection in early premature babies. Neurophysiol. Clin. 37, 299–309, 10.1016/j.neucli.2007.09.001 (2007). 18063232

[b64] Nunez P. L. . A theoretical and experimental study of high resolution EEG based on surface Laplacians and cortical imaging. Electroencephalogr. Clin. Neurophysiol. 90, 40–57 (1994). 7509273 10.1016/0013-4694(94)90112-0

[b65] Sanes J. R. & Yamagata M. Many paths to synaptic specificity. Annu. Rev. Cell Dev. Biol. 25, 161–195, 10.1146/annurev.cellbio.24.110707.175402 (2009). 19575668

[b66] Verriotis M. . Cortical activity evoked by inoculation needle prick in infants up to one-year old. Pain 156, 222–230, 10.1097/01.j.pain.0000460302.56325.0c (2015). 25599443 PMC4309489

