# Supplementary material for: Encoding of mechanical nociception differs in the adult and infant brain
Source: Sci Rep. 2016 Jun 27;6:28642. doi: 10.1038/srep28642 (PMC4921818; doi:10.1038/srep28642)
Supplement: Supplementary Information [file srep28642-s1.pdf]

Encoding of mechanical nociception differs in the adult and infant brain

Lorenzo Fabrizi*, Madeleine Verriotis, Gemma Williams, Amy Lee, Judith Meek[3], Sofia Olhede[2], Maria Fitzgerald.

Department of Neuroscience, Physiology & Pharmacology and [2] Department of Statistical Science, University College London, Gower Street, London WC1 E6BT, UK; [3] Elizabeth Garrett Anderson Obstetric Wing, University College Hospital, London NW1 2BU, UK

***Corresponding/Submitting Author**: Dr Lorenzo Fabrizi, Department of Neuroscience, Physiology & Pharmacology, University College London, Gower Street, London WC1 E6BT. Tel. +442031081888; E-mail l.fabrizi@ucl.ac.uk

Supplementary Methods

Time-frequency median estimator and group comparison

The time-frequency energy changes in the EEG that were induced by the stimuli were estimated as a group median because only one trial per subject was available. This was done by calculating the energy $\left|W^{(k)}(a,b)\right|^2$ of the TF transform for each individual trial k and then taking the sample median $\hat{S}_{r_x}(a,b)$ at each time-frequency point (a,b) for each group of trials r_x: (r_{al}) adult lance; (r_{ac}) adult control; (r_{il}) infant lance; (r_{ic}) infant control. $\hat{S}_{r_x}(a,b)$ was then normalised by the mean energy content $\hat{\sigma}^2_{r_x}(a)$ of the baseline period (-1000 to -500 ms before stimulus) at each frequency a. This represents the estimated percentage energy changes $\tilde{S}_{r_x}(a,b)$ phase- or non-phase-locked to the stimulus. We chose a median instead of a mean estimator to make the estimation less susceptible to the presence of outliers, but this meant that we could not use standard statistical tests and needed to derive the correct modelling distribution from first principles. Therefore we first determined the distribution for $\tilde{S}_{r_x}(a,b)$ and then for the ratio T between two $\tilde{S}_{r_x}(a,b)$ which was used to compare two different groups of trials.

To test the significance of the energy changes time-locked to the stimulus, we need to find the cumulative distribution function (CDF) of $\tilde{S}_{r_x}(a,b)$ as described elsewhere [1], but substituting the mean estimator with a median estimator. Considering, under the null hypothesis, the observed EEG signal, and therefore its wavelet transform, as Gaussian processes, it follows that:

$$U^{(k)} = \frac{\left|W^{(k)}(a,b)\right|^2}{\sigma^2(a)} \sim \frac{1}{2}\chi^2_2 \quad \forall k \tag{1}$$

The median $\tilde{S}$ of a sample of N trials taken from this distribution (i.e.$U^{(N/2)}$, assuming N *even*) will have the following probability density function (pdf):

$$f_{\tilde{S}} = f_{U^{(N/2)}}(u) = \frac{N!}{\left(\frac{N}{2}-1\right)!\left(\frac{N}{2}\right)!}(1-e^{-u})^{\frac{N}{2}-1}\ (e^{-u})^{\frac{N}{2}}\ e^{-u} =$$
$$= \frac{N!}{\left(\frac{N}{2}-1\right)!\left(\frac{N}{2}\right)!}(1-e^{-u})^{\frac{N}{2}-1}\ e^{-\left(\frac{N}{2}+1\right)u},\quad u > 0 \qquad (2)$$

Similarly, if N is *odd*, we obtain that:

$$f_{\tilde{S}} = f_{U^{\left(\frac{N+1}{2}\right)}}(u) = \frac{N!}{\left(\frac{N+1}{2}-1\right)!\left(N-\frac{N+1}{2}\right)!}(1-e^{-u})^{\frac{N+1}{2}-1}\ e^{-\left(\left(N-\frac{N+1}{2}\right)+1\right)u},\quad u > 0 \qquad (3)$$

The expressions (2) and (3) for N *even* or *odd* are similar only with different parameter choices. Integrating these expressions to calculate the CDF of $\tilde{S}_{r_x}(a,b)$ leads to:

$$F_{U^{(N/2)}}(u) = 1 - I_{e^{-u}}(N/2+1, N/2), \qquad (4)$$

which is an incomplete Beta function and can be used to perform the two-sided test to determine whether the energy changes $\tilde{S}_{r_x}(a,b)$ are significant.

In order to confront the patterns of evoked activity between adults and infants or between lance and control, we conducted group comparisons by calculating the ratio T between two $\tilde{S}_{r_x}(a,b)$. The ratio $T_{r_{al}r_{ac}} = \frac{\tilde{S}_{r_{al}}}{\tilde{S}_{r_{ac}}}$ represents the proportional difference between lance and control stimulation in adults; $T_{r_{il}r_{ic}} = \frac{\tilde{S}_{r_{il}}}{\tilde{S}_{r_{ic}}}$ between lance and control in infants; $T_{r_{il}r_{al}} = \frac{\tilde{S}_{r_{il}}}{\tilde{S}_{r_{al}}}$ between infant and adult lance and $T_{r_{ic}r_{ac}} = \frac{\tilde{S}_{r_{ic}}}{\tilde{S}_{r_{ac}}}$ between infant and adult control. To test for the significance of these differences, we need to obtain the pdf and cumulative distribution function (CDF) of the ratio $T_{r_1r_2}$ (comparing group 1 and 2):

$$T_{r_1r_2}(a,b) = \frac{\tilde{S}_{r_1}(a,b)}{\tilde{S}_{r_2}(a,b)} = \frac{U_{r_1}}{U_{r_2}}, \qquad (5)$$

which is the ratio between two random variables of the form of (4). These are unpaired comparisons because the number of trials, even within the same age group, was different.

The CDF of (5) is computed using a variables substitution and numerical integration. From first principles using transformation methods, the joint pdf of Y_1 and Y_2 is:

$$f_{Y_1Y_2}(y_1, y_2) = |J| \cdot f_{U_{r_1}}(y_1y_2) \cdot f_{U_{r_2}}(y_2) \quad (6)$$

where J is the Jacobian of the transformation.

The marginal distribution of Y_1 is therefore given by the numerical integration:

$$f_{Y_1}(y_1) = \int_0^{\infty} f_{Y_1Y_2}(y_1, y_2)dy_2 \quad (7)$$

In order to test for the significance of $T_{r_1r_2}(a, b)$ we need to conduct a second numerical integration along y_1, yielding to the CDF of $Y_1 = \frac{U_{r_1}}{U_{r_2}}$:

$$F_{Y_1}(y_1) = \int_0^{y_1} f(y_1)dy_1 \quad (8)$$

The significance of the tests for the CDF in equation (4) and (8) was tried against a threshold of α = 0.05, however, because these tests were conducted at any point (a, b) of the time-frequency plane, false discovery rates was used to correct for multiple comparisons. The total number of independent tests performed was estimated in two steps: (i) calculating the number of independent tests at each frequency by dividing the length of the considered epoch by the length of the wavelet at that frequency (accounting for the correlation caused by the smoothing in time of the wavelet transform); (ii) adding those numbers across frequencies.

References

1 Fabrizi, L. *et al.* Cortical activity evoked by an acute painful tissue-damaging stimulus in healthy adult volunteers. *J. Neurophysiol.* **109**, 2393-2403, doi:10.1152/jn.00990.2012 (2013).

Supplementary Figure 1

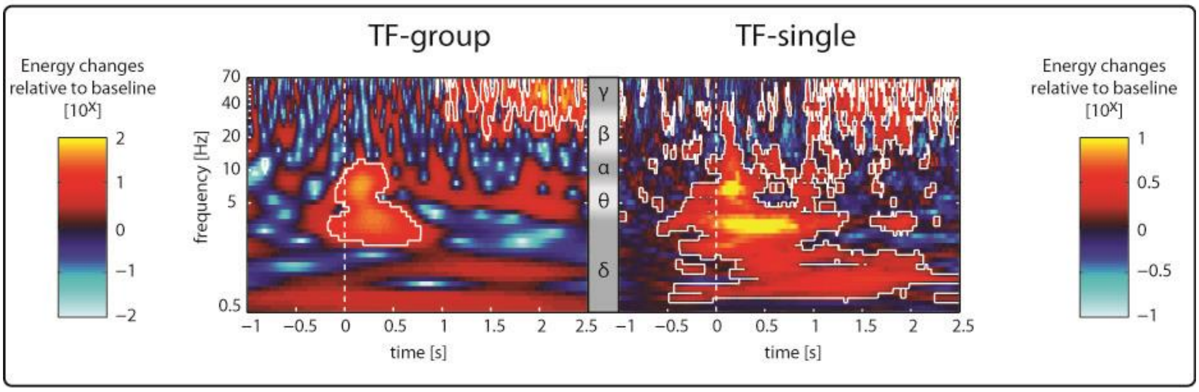

Supplementary Figure 1. Group and single time-frequency (TF) transform of the EEG response at Cz to a skin-breaking noxious stimulus in full term infants. TF-single is obtained by calculating the TF transform for each individual trial and then taking the median in the time-frequency domain as described in the main text. TF-group, instead, is obtained by calculating the TF transform of the averaged trace. EEG changes present in both representations should be considered as being phase-locked across trials, while those that are only present in TF-single should be considered as non-phase-locked. The fast delta (2-4 Hz) nociceptive and age specific activity is non-phase locked between 750 and 1300 ms, while the late gamma activity is largely phase-locked.
